# Vitamin D and Calcium Intakes, Physical Activity, and Calcaneus BMC among School-Going 13-Year Old Malaysian Adolescents

**DOI:** 10.3390/nu8100666

**Published:** 2016-10-24

**Authors:** A. A. Suriawati, Hazreen Abdul Majid, Nabilla Al-Sadat, Mohd Nahar Azmi Mohamed, Muhammad Yazid Jalaludin

**Affiliations:** 1 Centre for Population Health (CePH), Department of Social and Preventive Medicine, Faculty of Medicine, University of Malaya, Kuala Lumpur 50603, Malaysia; misssuriya@yahoo.co.uk (A.A.S.); hazreen@ummc.edu.my (H.A.M.); nabilla@ummc.edu.my (N.A.-S.); 2 Department of Nutrition, Harvard T.H. Chan School of Public Health, Boston, MA 02115, USA; 3 Sports Medicine Unit, Faculty of Medicine, University of Malaya, Kuala Lumpur 50603, Malaysia; nahar@ummc.edu.my; 4 Department of Paediatrics, Faculty of Medicine, University of Malaya, Kuala Lumpur 50603, Malaysia

**Keywords:** vitamin D, calcium, adolescents, bone mineral content (BMC), MyHeARTs

## Abstract

Background: Dietary calcium and vitamin D are essential for bone development. Apart from diet, physical activity may potentially improve and sustain bone health. Objective: To investigate the relationship between the dietary intake of calcium and vitamin D, physical activity, and bone mineral content (BMC) in 13-year-old Malaysian adolescents. Design: Cross-sectional. Setting: Selected public secondary schools from the central and northern regions of Peninsular Malaysia. Participants: The subjects were from the Malaysian Health and Adolescents Longitudinal Research Team Cohort study (MyHeARTs). Methods: The data included seven-day diet histories, anthropometric measurements, and the BMC of calcaneal bone using a portable broadband ultrasound bone densitometer. Nutritionist Pro software was used to calculate the dietary calcium and vitamin D intakes from the diet histories, based on the Nutrient Composition of Malaysian Food Database guidance for the dietary calcium intake and the Singapore Energy and Nutrient Composition of Food Database for vitamin D intake. Results: A total of 289 adolescents (65.7% females) were recruited. The average dietary intakes of calcium and vitamin D were 377 ± 12 mg/day and 2.51 ± 0.12 µg/day, respectively, with the majority of subjects failing to meet the Recommended Nutrient Intake (RNI) of Malaysia for dietary calcium and vitamin D. All the subjects had a normal Z-score for the BMC (−2.00 or higher) with a mean of 0.55 ± 0.01. From the statistical analysis of the factors contributing to BMC, it was found that for those subjects with a higher intake of vitamin D, a higher combination of the intake of vitamin D and calcium resulted in significantly higher BMC quartiles. The regression analysis showed that the BMC might have been influenced by the vitamin D intake. Conclusions: A combination of the intake of vitamin D and calcium is positively associated with the BMC.

## 1. Introduction

Childhood and adolescence are crucial periods for skeletal mineralization. Bone mass accumulates in the skeleton, and bone density generally reaches its peak level in late adolescence [1]. This is a critical step towards achieving maximal peak bone mass early in life [2]. Hence, the risk of getting osteoporosis or low bone mass in later life can be reduced [3]. Sixty percent of the variance in peak bone mass is determined genetically. However, the remainder is most likely influenced by factors that are amenable to positive interventions, such as vitamin D, physical activity, and an adequate dietary intake of dairy products as a natural source of calcium [4]. The intake of vitamin D (a lipid-soluble vitamin) is essential for calcium absorption and utilization, and for mineral metabolism for bone development and health during adolescence [5,6,7]. There have been reports suggesting that vitamin D deficiency is common worldwide, especially in the western world, both among adults and adolescents [8,9,10]. It is necessary to increase the intake of calcium during adolescence, as it is an essential bone-forming mineral for optimal growth and development, where it can affect the acquisition of bone mass. About 45% to 50% of the skeletal mass is accumulated during adolescence [11,12,13]. It has been found that physical activity, together with nutrition, contributes to bone mass during growth [2,14,15]. Systematic reviews have found that these modifiable factors are beneficial for bone mass and density [3,16]. As far as is known, no study has been performed to date on the Asian adolescent population to address the influence of dietary vitamin D, calcium, physical activity, and BMC on the calcaneus.

Although various studies and reviews relating nutrition to bone health in adolescents have been conducted elsewhere [6,17,18,19], the research conducted in Malaysia has been limited. In the South-East Asian Nutrition Survey (SEANUTS)—a collaborative study of Indonesia, Malaysia, Thailand, and Vietnam of 6 months to 12-year-old infants and children—measurement of the bone mineral content (BMC) of the tibia and/or the radius of the Malaysian participants was taken using a quantitative ultrasound technique [20]. In the Malaysian NHMS (National Health and Morbidity Survey) in 2012, only the dietary calcium intake among subjects aged 10 to 17 years was assessed [21], but not their dietary intake of vitamin D. A study on the prevalence of vitamin D deficiency involving 402 boys and girls aged 7 to 12 years attending primary schools in Kuala Lumpur also did not assess the dietary vitamin D intake [22]. Most of the bone studies among adolescents did not assess the dietary intake, thereby limiting further analyses on the correlation between dietary intake and serum micronutrient status [22,23]. However, in this study, a validated seven-day diet history tool was used to estimate the actual habitual dietary intake among adolescents [24,25,26,27].

To date, this is the first cross-sectional study investigating the association between dietary calcium and vitamin D with BMC among adolescents in Malaysia. Through this study, healthcare professionals may gain some insight into the nutritional status of adolescents in Malaysia so that interventions can be planned accordingly.

## 2. Materials and Methods

### 2.1. Subjects

This study used the baseline data gathered from 13-year old adolescent schoolchildren from public schools under the Malaysian Health and Adolescents Longitudinal Research Team Cohort study (MyHeARTs). MyHeARTs is an on-going prospective cohort study using a multistage cluster sampling design. The subjects were adolescents from government schools, with the exception of boarding, religious, and vernacular schools. The study protocol was published [28] and approved by the Medical Ethics Committee, University Malaya Medical Centre (MEC Ref. No.: 896.34), and the National Medical Research Register number is 14-376-20486. Informed consent was obtained in writing prior to the data collection. A number of schools from urban and rural locations were randomly selected based on the criteria provided by the Department of Statistics, Malaysia, using computer-generated random number lists. These schools were from Kuala Lumpur, Putrajaya, and Perak, and were recommended by the Ministry of Education, Malaysia. The consent of parents or guardians was obtained. Only samples with complete data on BMC and the dietary intake of calcium and vitamin D were included in the analysis.

This analysis, which involved the first wave of the MyHeARTs cohort, was held from March to May 2012. It excluded under- and over-reporting on the diet of the subjects. Therefore, the final sample used in this article included 289 participants (65.7% females).

### 2.2. Anthropometric Measurements

All of the anthropometric measurements were done by trained personnel. The subjects were required to empty their bladder before their body measurements were taken. The weights of the subjects were measured using a digital electronic weighing scale (Seca 813, Seca, Birmingham, UK) and were recorded to the nearest decimal fraction of a kilogram (0.1 kg). The heights of the subjects without socks and shoes were measured to the nearest 0.1 cm using a calibrated vertical stadiometer (Seca Portable 217, Seca, Birmingham, UK). The body fat was analysed using a portable body composition analyser (Tanita SC- 240 MA Portable Body Composition Analyser, Tanita Europe B.V., Amsterdam, The Netherlands). Before the measurements were taken, the participants were asked to wear light clothing, remove all their accessories and socks, and empty their pockets. In this analysis, the body mass index (BMI) was calculated by dividing the participant’s weight (in kilograms) by the square of the participant’s height (in meters). The BMI reference used was the International Obesity Task Force (IOTF) classification 2000, an international data reference produced on children in the US, UK, Hong Kong, the Netherlands, Singapore, and Brazil with regard to gender, and specifically for ages 2–18 years. The IOTF cut-off values for the BMI are linked to the adult cut-off values of 25 and 30 for risk of overweight and obesity, respectively, for adolescents aged 18 years [29,30,31].

### 2.3. Dietary Assessment

Generally, habitual dietary intake can be assessed using several methods, such as diet histories (24-h or seven-day dietary recall), food frequency, three days’ dietary records, and duplicate food weighing to evaluate energy intake. However, the seven-day diet history was chosen for this study, as this method has produced more valid estimates of energy intake in children and adolescents compared to other methods [24,32]. The tool was pretested on 40 participants from two different schools (one school each from an urban and rural area, respectively). For the actual study, seven qualified and trained dieticians used open-ended interviews with the students to collect information on the food and drinks that they consumed for breakfast, mid-morning snacks, lunchtime, afternoon tea, dinner, and supper over the previous seven-day period. Illustrated flip charts of local food were used as supplementary tools to assist the study participants during the dietary evaluation and to help estimate the portion size of the food portions that they consumed [33]. The intake of nutrients was calculated using the Nutritionist Pro™ Diet Analysis (Axxya Systems, Woodinville, WA, USA) software with the Nutrient Composition of Malaysian Food Database guidance for the dietary calcium intake and the Singapore Energy and Nutrient Composition of Food Database [34] for the vitamin D intake, as the Malaysian Food Database has a calcium component but not a vitamin D component. Since Singapore is geographically nearer and has food that is almost similar to the food in Malaysia, its food database was therefore chosen for analysis of the vitamin D intake.

To determine and correct for underreported dietary intake, the ratio of the energy intake to the predicted basal metabolic rate (EI:BMR) was calculated. The basal metabolic rate (BMR) was calculated using the Henry and Rees equations (1991) [35], as these were built on a database of mixed populations that included persons from tropical regions, which is similar to the mixed population and climate in Malaysia. The EI:BMR ratio was calculated by dividing the average energy intake by the BMR. Then, the ratio that was obtained was compared with the cut-off point set by Kersting et al. [36]. Those ratio values that were lower than the cut-off point were excluded from the data analysis.

### 2.4. Pubertal Stage

The pubertal stages were self-assessed using coloured illustrations of the Tanner stages. The rating for male sexual maturity was based on the development of external genitalia and pubic hair, while the rating for female sexual maturity depended on the development of breasts and pubic hair. A numerical scale of 1 to 5 was used for the different stages of pubertal development (i.e., from stage 1 to stage 5).

### 2.5. Bone Measurement

In this study, the measurement of the bone mineral content (BMC) was made at the calcaneus. According to the International Society of Clinical Densitometry (ISCD), a calcaneal quantitative ultrasound (QUS) can be used as a reference for the total BMC [37]. According to the official position statement released by ISCD 2007, the calcaneal QUS is the only recognized measurement of QUS that has been validated for clinical use as the determinant of the status of bone health [38]. A QUS at the calcaneus is useful and can provide information on the bone mineral status and the risk of fracture, as reported by Guglielmi et al. [39], Khaw et al. in the European Prospective Investigation into Cancer (EPIC)-Norfolk prospective population study of British male and female populations [40], and Krieg et al. in the prediction of risk of a hip fracture at different QUS sites [41]. This has also been supported by studies which found the calcaneus QUS to be effective in predicting the risk of fracture [42,43]. Therefore, it should be used for the screening of osteoporosis, especially in developing countries such as Malaysia, where dual-X-ray absorptiometry devices are less accessible to the general population [44].

BMC of the left calcaneus was measured using a portable broadband ultrasound bone densitometer (Hologic Sahara, Hologic Inc., Marlborough, MA, USA, version 3.0.1). After a comparison was made to the appropriate normal control, the reported value was given as a percentile or a standard deviation score (known as a Z-score) for the adolescents.

### 2.6. Physical Activity Level

A self-reporting questionnaire on the physical activity over the previous week was assessed using a Malay version of a validated physical activity questionnaire for older children (PAQ-C), which had good internal consistency and acceptable validity, as described in previous studies [45,46,47]. Ten items in the PAQ-C were designed to capture the level of physical activity seven days prior to filling in the questionnaire. The mean self-reported physical activity score levels were further categorized into low (score < 2.33), moderate (2.33–3.66), and high (score > 3.66) [45,46,47]. The first item in the questionnaire included the type and frequency of sports or/and dances that the subjects participated in during the past seven days. The second to the eighth items in the questionnaire assessed the level of activity of the adolescents during physical education (PE) classes, recess, at lunchtime, immediately after school, in the evenings, weekend, and leisure time. The answers to items two to eight were based on a five-point Likert scale (1 (lowest) to 5 (highest)). Item nine included the frequency of participation in a daily physical activity in the previous week. Item ten asked the subjects to report any unusual activities during the previous week which had not been recorded.

### 2.7. Statistical Analysis

The Statistical Package for Social Sciences version 22.0 (IBM Corp., Armonk, NY, USA) was used to analyse the data. A univariate analysis for complex samples (CS) was performed on the weighted data. A general linear model (GLM) in the CS determined the associations between the BMC (continuous variable) and dietary vitamin D intake, dietary calcium intake, and physical activity, adjusted for gender and puberty. The values presented the adjusted B values (estimated unstandardized regression coefficient) and the standard errors. All the statistical tests and corresponding *p*-values were two-sided, and a *p*-value less than 0.05 was considered statistically significant.

The continuous data on the calcium intake (mg/day), vitamin D intake (µg/day), and physical activity (score) were transformed into quartiles of categorical data for a further regression analysis in one statistical analysis between the factors. The tested factors of the dietary vitamin D and calcium intakes were combined in the same statistical analysis to become another factor that was later known as a combination of vitamin D and calcium intakes. This was done by using a regression analysis within a statistical analysis to show the relevant relationship between the vitamin D and calcium intakes when they were taken into account together from the same data composition of each person in the study. The combination of vitamin D and calcium intakes was constructed by a statistical analysis in SPSS using a GLM, where the interactions between the independent variables (calcium intake and vitamin D intake) were calculated and tested to determine whether they were statistically related to the dependent variable, the BMC. In this analysis, adjustments were made for gender and the pubertal stage.

## 3. Results

### 3.1. Descriptive Characteristics of Male and Female Subjects

Table 1 shows the descriptive characteristics of the subjects in the study according to gender. The females had a higher BMC (*p* < 0.05), body weight, BMI, and dietary calcium intake tendency compared to the males, but a lower vitamin D intake tendency and physical activity score. It was found that the BMC tendency was higher in those with a moderate physical activity score of 0.55 ± 0.01 compared to those with a low physical activity score (0.54 ± 0.01) with no significant effect. However, in this study, none of the subjects fulfilled the criteria to be categorized as active.

### 3.2. Sample Characteristics According to Puberty Level

After the children had been classified into the pre-pubertal and pubertal groups (as shown in Table 2), it was found that the BMC of the pubertal subjects was higher than that of the pre-pubertal subjects. Both groups were unable to meet calcium and Vitamin D Recommended Nutrient Intake (RNI) for Malaysians, which is 1000 mg per day and 5 μg per day, respectively. All the subjects showed low levels of physical activity with no significant differences, although it was found that the pre-pubertal subjects had a slightly higher tendency to engage in physical activity.

### 3.3. Quartile Values

Table 3 displays the quartile value for each factor of analysis in this study that contributed to the BMC. A quartile is a value when numerical data are divided and converted into four segments of categorical data. Q1 was the lowest and Q4 was the highest value for the Calcium intake (mg/day), Vitamin D intake (µg/day), and Physical Activity (score) parameters.

### 3.4. Regression Analysis

To investigate whether the BMC was influenced by calcium intake, vitamin D intake, a combination of vitamin D and calcium intakes, and physical activity, a general linear model via CS analysis was performed to test the dependency of the BMC (Table 4) on quartiles of calcium intake, vitamin D intake, and physical activity, controlled for gender and pubertal stage. Positive associations were found in the quartiles of vitamin D intake and with a combination of vitamin D and calcium intakes. However, in this model, the quartiles of calcium intake and physical activity did not show any relationship with the BMC.

## 4. Discussion

In Table 1, the BMC was found to be higher in females compared to the male subjects, perhaps due to a higher body weight (*p* < 0.01). A higher body weight may influence the BMC through the mechanical loading factor from the increased body weight [48]. A systematic review found that low body weight is one of the risk factors for fractures [49]. A study by Yilmaz et al. demonstrated that weight is one of the determinants of bone mass in adolescents [50]. Bone growth in males is different from female adolescents, where the onset is later by two years compared to females (at age 14 compared to 12 in females), and the male pubertal growth spurt lasts for an additional year compared to only three years in females [51]. This could be one of the factors contributing to the differences in BMC between male and female adolescents. There was a tendency for the calcium intake to be higher in females, but with no significant differences.

Most of the subjects (98.8% and 91.7%) were unable to meet Recommended Nutrient Intake (RNI) for dietary calcium and vitamin D, respectively in Malaysia (1000 mg/day for calcium and 5 µg/day for vitamin D). Their calcium intake was lower compared to that of Europeans in a previous study on calcium inadequacy [52,53,54]. In Germany, 80% of children aged 1 to 12 years were unable to meet the 5 µg/day vitamin D daily requirement as recommended by the German Society for Nutrition. The Dortmund Nutritional and Anthropometric Longitudinally Designed Study (DONALD) [55] and Eating Study as a German Health Interview and Examination Survey for Children and Adolescents Module (EsKiMo) [56] studies found that the vitamin D intake for adolescents was 2.5 µg/day in males and 1.9 µg/day in females. It was found that the mean value of vitamin D intake in this study was slightly higher compared to the EsKiMo study, where the intake was 2.55 µg/day in males and 2.50 µg/day in females, but was lower compared to an earlier study on Finnish girls [57], where their intake was 4.3 µg/day. A recent study found that the mean vitamin D intake from both food and supplements for Finnish children was 5.9 µg/day in males and 7.7 µg/day in females [58]. Even though female adolescents have higher BMC value compared to male adolescents, it has been found that the male adolescents have a normal mean BMC level but with a tendency for higher vitamin D intake and higher physical activity levels (*p* = 0.001) compared to females. This study demonstrated that the diet of male adolescents consists of more food that is high in vitamin D content compared to females. Nevertheless, it is still unable to meet the Malaysian RNI, as there is a limited number of foods fortified with vitamin D in Malaysia. Apart from dietary intake, vitamin D can be produced by exposing human skin to UV-B radiation in the range of 290 to 315 nm [11]. According to its latitude, Malaysia is considered as a sunny country, where the sun shines throughout the year. This was the reason why the production of vitamin D as a result of skin synthesis was not measured in the first wave of the MyHeART study.

The 2014 clinical report of the American Academy of Paediatrics mentioned that hormones play a role in promoting bone formation in healthy adolescents [11]. These hormones include growth hormone and insulin-like growth factor-I (IGF-I). In a study of the young healthy men, it was found that the BMC is positively correlated with the magnitude of the secretion of the growth hormone in young healthy men in the United States [59]. This may explain why the BMC of males is normal, as it may be influenced by hormones.

From Table 2 it was discovered that the BMC was higher in the pubertal group compared to the pre-pubertal group, with significant differences (*p* = 0.01), and that both groups had normal BMC values. Puberty plays a fundamental role in the acquisition of peak bone mass [51]. It is widely known that adolescents in the pubertal stage will have a higher bone mass compared to those in the pre-pubertal stage, as demonstrated in a study by Van Coeverden et al. [60]. A study by Yilmaz et al. [50] proved that bone mass increases throughout puberty in both genders. The substantial impact of puberty on bone is very well described in both boys and girls, where Arabi et al. reported an increase of up to 60% in bone mass in all skeletal parts at later Tanner stages [61].

Table 3 shows the quartile cut-off values that were used for the analysis in this study. The quartile data included the main factors that were considered to have been associated with BMC, such as calcium intake, vitamin D intake, and physical activity. The quartile for the combination of vitamin D and calcium intakes were further considered together in the next regression analysis, as displayed in Table 4. The quartile for the combination of vitamin D and calcium intakes was constructed using the statistical analysis of the GLM in SPSS to test the assumption of the relationship between both calcium and vitamin D intake together with the BMC.

To further assess relationships between the calcium intake, vitamin D intake, and physical activity with BMC, a GLM via CS was performed using quartile values with the controlling confounders of gender and puberty level. Table 4 demonstrates the relationship between the quartiles of vitamin D intake, calcium intake, the combination of vitamin D and calcium intakes, and physical activity, controlling for the gender of the subjects. The quartiles of vitamin D intake and the combination of vitamin D and calcium intakes that had positive correlations with the BMC accounted for 11.8% of the variance in BMC. The equation found from this study was BMC = 0.569 + (0.155 × quartiles vitamin D) + (0.199 × quartiles vitamin D and calcium intake). A previous study indicated that dietary calcium is one of the main determinants of BMD in children and adolescents [62]; and in a longitudinal study of Caucasian females, the intake of calcium—especially from dairy foods—was shown to have a favourable effect on whole-body BMC during middle childhood [63]. However, the results of the current study were in contrast to these findings, probably due to the limited number of subjects with adequate calcium intake within the sample. A study demonstrated that there was a positive relationship between spontaneous calcium intake and bone mass in a group of children with only high intake of calcium of around 1000 to 1200 mg/day [64]. Perhaps this was why this study did not show any significant relationship between calcium and BMC. A continuously adequate dietary calcium intake is necessary for calcium to have a positive effect of on bone mass. This was shown in a study where, upon discontinuing calcium supplementation, a follow-up after one-year showed no significant differences between the calcium-supplemented and the placebo-control groups [27,65]. Vitamin D plays a key role in calcium metabolism, and is essential for bone health in adolescents [6], where it is used for important bone development [7]. It has been revealed in this study that the intake of vitamin D had a positive relationship with the BMC, unlike a study among Spanish adolescents [66]. It was concluded in a systematic review that vitamin D supplementation in healthy children with deficient vitamin D levels may result in an improvement in the BMC [67]. A study by Picard et al., however, showed that the intake of vitamin D is an independent predictor of bone mass [68]. However, in this study, the combination of vitamin D and calcium intakes was shown to have a significant effect on the BMC.

This study was consistent with that of Molgaard et al. [64], where it was found that there is no significant association between the time spent on being engaged in physical activity and the BMC. This is maybe due to the lack of calcium intake among the subjects in the study, with the mean being below 1000 mg/day of the Malaysian RNI. According to Ruiz et al. [69], the association between physical activity and bone mass can only be found in those whose calcium intake is >800 mg/day. This is in agreement with a finding by Specker [70] where physical activity had no effect on the bone at a calcium intake of <1000 mg/day.

The strengths of this study are that it is the first study among adolescents in Malaysia that used the calcaneal BMC as a measurement, where it is only validated for clinical use for bone health status [38]; it is effective in predicting fracture risk [42,43], and it is more accessible to the general population in developing countries such as Malaysia [44]. This study included the calculated dietary vitamin D intake together with the calcium intake, where these were used to test the relationship with the BMC using a seven-day diet history. The seven-day diet history was conducted on a one-to-one basis by trained and certified dieticians. The dieticians used several tools to assist in their data collection: they used household measurement tools (plates, cups, glasses, bowls, ladle, spoons); a flip chart that illustrated common Malay, Chinese, and Indian foods, and a diet history collection tool. A 24-h diet recall alone would not have been able to capture the precise diet of each participant [26]. Another method for the assessment of dietary intake is the weighed record method, which is more expensive than the analysis of a group, and is not suitable for population-based studies [71]. This study had its limitations, in that there was no device to measure physical activity, the exposure to the sun was not measured, and the analysis did not include the concentrations of plasma 25-hydroxyvitamin D (25OHD). However, the self-reporting questionnaire (PAQ-C) that was used was able to evaluate the physical activity level of the subjects. In this study, the subjects were categorised according to their physical activity level, but not their energy expenditure. In the next wave of this study, the exposure to the sun will also be included. Most of the adolescent subjects in this study did not achieve the RNI for dietary calcium and vitamin D values, thereby limiting the results of the significant effects in the statistical analysis. However, this was overcome by the exclusion of missing or unreliable data. This study was a cross-sectional study, where the results could not be interpreted in terms of cause and effect. Data from longitudinal studies were required to clarify the relationship between the calcium intake, vitamin D intake, and the combination of calcium and vitamin D intakes, physical activity, and BMC.

## 5. Conclusions

A positive association was observed between the BMC and vitamin D intake, as well as with the combination of vitamin D and calcium intake. Further research on the effect of other factors in addition to diet (such as exposure to the sun) should be undertaken in a longitudinal study, so that the cause–effect relationship can be determined.

## Figures and Tables

**Table 1:** Characteristics of participants according to gender (*n* = 289).

Characteristics	All ^a^ (*n* = 289)	Male ^a^ (*n* = 99, 34.23%)	Female ^a^ (*n* = 190, 65.7%)	*p-*Value ^b^
Height (cm)	150.8 ± 0.41	150.1 ± 0.89	151.1 ± 0.44	NS
Weight (kg)	42. 6 ± 0.64	40.3 ± 1.04	43.7 ± 0.80	0.010 (S)
BMI (kg/m^2^)	18.65 ± 0.24	17.78 ± 0.36	19.08 ± 0.31	0.006 (S)
Calcium intake (mg/day)	377 ± 12	356 ± 16	387 ± 16	NS
Vitamin D intake (µg/day)	2.51 ± 0.12	2.55 ± 0.23	2.50 ± 0.14	NS
BMC (Z-score)	0.55 ± 0.01	0.52 ± 0.01	0.56 ± 0.01	0.004 (S)
Physical Activity (Score)	2.01 ± 0.03	2.18 ± 0.06	1.93 ± 0.04	0.001 (S)

^a^ Data expressed as mean ± standard error; ^b^ *p* < 0.05 (two-tailed); S—significant and NS—not significant. BMC: bone mineral content; BMI: body mass index.

**Table 2:** Characteristics according to puberty level (*n* = 289).

Characteristics	All^a^ (*n* = 289)	Pre-Pubertal ^a^ (*n* = 21, 0.1%)	Pubertal ^a^ (*n* = 268, 99.9%)	*p-*Value ^b^
Height (cm)	150.8 ± 0.41	146.9 ± 1.32	151.0 ± 0.42	0.004 (S)
Weight (kg)	42. 6 ± 0.64	41.4 ± 2.68	42.6 ± 0.66	NS
BMI (kg/m^2^)	18.65 ± 0.24	19.1 ± 1.11	18.6 ± 0.31	NS
Calcium intake (mg/day)	377 ± 12	414 ± 35	375 ± 12	NS
Vitamin D intake (µg/day)	2.51 ± 0.12	2.39 ± 0.28	2.52 ± 0.13	NS
BMC (Z-score)	0.55 ± 0.01	0.46 ± 0.02	0.56 ± 0.01	0.001 (S)
Physical Activity (score)	2.01 ± 0.03	2.18 ± 0.18	1.20 ± 0.03	NS

^a^ Data expressed as mean ± standard error; ^b^ *p* < 0.05 (two-tailed); S—significant and NS—not significant.

**Table 3:** Quartile values of dietary calcium, vitamin D, and physical activity.

Parameter	Q1 ^a^	Q2 ^a^	Q3 ^a^	Q4 ^a^
Calcium intake (mg/day)	207 ± 5	350 ± 6	581 ± 10	926 ± 55
Vitamin D intake (µg/day)	0.85 ± 0.04	2.17 ± 0.04	3.71 ± 0.07	5.68 ± 0.22
Physical activity (score)	1.49 ± 0.01	1.79 ± 0.01	2.11 ± 0.01	2.66 ± 0.01

^a^ Data expressed as mean ± standard error; Q1—quartile 1, Q2—quartile 2, Q3—quartile 3 and Q4—quartile 4.

**Table 4:** Associations between BMC and quartiles of dietary calcium, vitamin D, and physical activity of individuals in the Malaysian Health and Adolescents Longitudinal Research Team Cohort study (MyHeARTs) (unstandardized parameter estimates).

Characteristics	BMC (Z-Score) *R*^2^ = 0.156		
	B ^a^	SE	*p-*Value ^b^
Quartiles of vitamin D intake (µg/day)	0.794	0.054	0.028 (S)
Quartiles of calcium intake (mg/day)	0.516	0.089	NS
Quartiles of combination of vitamin D (µg/day) and Calcium intake (mg/day)	0.203	0.070	0.020 (S)
Quartiles of physical activity (scores)	0.639	0.036	NS

Abbreviation: BMC, bone mineral content. All data adjusted for gender and puberty. All variables were in quartiles. ^a^ B is the estimated unstandardized regression coefficient; ^b^ Significant, *p* < 0.05; NS—not significant.

## Abbreviations

The following abbreviations are used in this manuscript:

BMI	Body Mass Index
BMC	Bone Mineral Content
BMD	Bone Mineral Density
BMR	Basal Metabolic Rate
CS	Complex Sample
EI:BMR	The ratio of the energy intake to the predicted basal metabolic rate
FFQ	Food Frequency Questionnaire
GLM	General Linear Model
ISCD	International Society of Clinical Densitometry
MyHeARTs	The Malaysian Health and Adolescents Longitudinal Research Team Cohort study
PBM	Peak Bone Mass
PAQ-C	Physical Activity Questionnaire for older Children
QUS	Calcaneal Quantitative Ultrasound
RNI	Recommended Nutrient Intake
UM	University of Malaya
UV	Ultra Violet
25OHD	25-hydroxyvitamin D
